# Nuclear microsatellite and mitochondrial DNA analyses reveal the regional genetic structure and phylogeographical history of a sanguivorous land leech, *Haemadipsa japonica*, in Japan

**DOI:** 10.1002/ece3.5132

**Published:** 2019-04-03

**Authors:** Kaori Morishima, Mineaki Aizawa

**Affiliations:** ^1^ United Graduate School of Agricultural Science Tokyo University of Agriculture and Technology Utsunomiya Japan; ^2^ Department of Forest Science School of Agriculture Utsunomiya University Utsunomiya Japan

**Keywords:** genetic diversity, genetic drift, mitochondrial DNA lineage, northward migration, nuclear microsatellite

## Abstract

Recent molecular studies have indicated that phylogeographical history of Japanese biota is likely shaped by geohistory along with biological events, such as distribution shifts, isolation, and divergence of populations. However, the genetic structure and phylogeographical history of terrestrial Annelida species, including leech species, are poorly understood. Therefore, we aimed to understand the genetic structure and phylogeographical history across the natural range of *Haemadipsa japonica*, a sanguivorous land leech species endemic to Japan, by using nine polymorphic nuclear microsatellites (nSSR) and cytochrome oxidase subunit one (COI) sequences of mitochondrial DNA (mtDNA). Analyses using nSSR revealed that *H*. *japonica* exhibited a stronger regional genetic differentiation among populations (*G*'_ST_ = 0.77) than other animal species, probably because of the low mobility of land leech. Analyses using mtDNA indicated that *H*. *japonica* exhibited two distinct lineages (A and B), which were estimated to have diverged in the middle Pleistocene and probably because of range fragmentation resulting from climatic change and glacial and interglacial cycles. Lineage A was widely distributed across Japan, and lineage B was found in southwestern Japan. Analyses using nSSR revealed that lineage A was roughly divided into two population groups (i.e., northeastern and southwestern Japan); these analyses also revealed a gradual decrease in genetic diversity with increasing latitude in lineage A and a strong genetic drift in populations of northeastern Japan. Combined with the largely unresolved shallow polytomies from the mtDNA phylogeny, these results implied that lineage A may have undergone a rapid northward migration, probably during the Holocene. Then, the regional genetic structure with local unique gene pools may have been formed within each lineage because of the low mobility of this leech species.

## 1 INTRODUCTION

**Figure 1:**
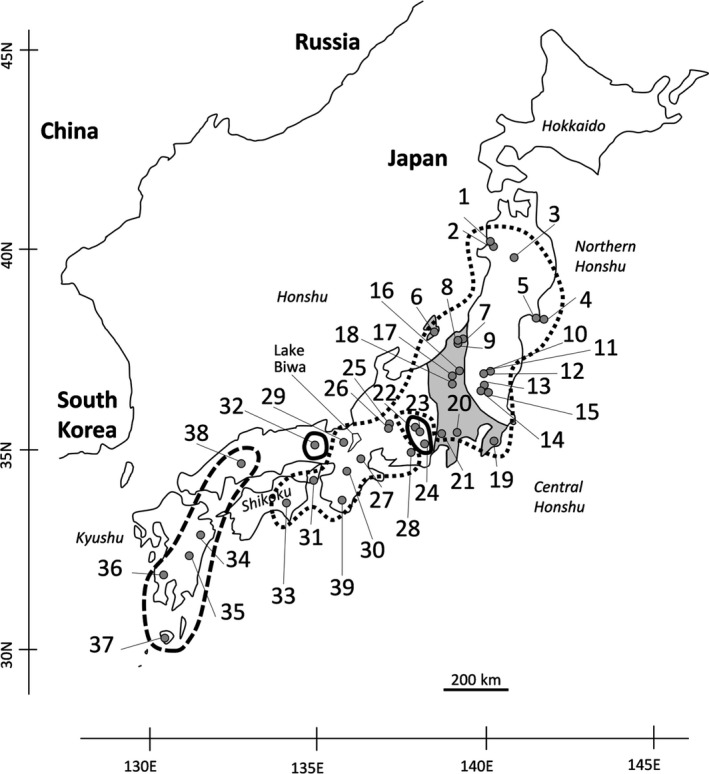
Sampling locations for the genetic analyses. Population codes (1–39) correspond to those shown in Table 1. Populations within areas surrounded by dotted, thin, and dashed lines harbored mitochondrial DNA (mtDNA) lineage A, sublineage B1, and sublineage B2, respectively. The area in gray indicates the Fossa Magna region in central Honshu

The Japanese archipelago comprises four main islands (Hokkaido, Honshu, Shikoku, and Kyushu; Figure 1) and is located off the far eastern coast of the Asian continent. The landmasses that later formed the Proto‐Japanese Islands were originally located along the eastern fringe of the Asian continent. Approximately 15 million years ago (Mya), two landmasses—the northeastern and southwestern portions of the Proto‐Japanese Islands—began to independently separate from the Asian continent, along with the formation of the Sea of Japan. Before the two landmasses were connected to each other, eastern and western Japan were separated by a sea zone (channel) called the Fossa Magna, which longitudinally traversed central Honshu, Japan (area in gray; Figure 1), during the Miocene (around 15–5 Mya; Tojo, Sekiné, Suzuki, Saito, & Takenaka, 2017). The Fossa Magna was later filled with thick sediments from the Tertiary (Takeda et al., 2004), and the subsequent elevation of the surrounding land during the Quaternary led to the formation of the high mountain chains in central Honshu. The Fossa Magna and orogenic movements are considered important dispersal barriers for many animal species; recent molecular phylogeographical analyses have indicated deep divergences between inter‐ and intraspecific vicariants in the Fossa Magna region (Sekiné, Hayashi, & Tojo, 2013; Shoda‐Kagaya et al., 2010; Suzuki, Sato, & Ohba, 2002; Tojo et al., 2017; Watanabe et al., 2006; Watanabe, Tominaga, Nakajima, Kakioka, & Tabata, 2017). Furthermore, glacial and interglacial cycles have also been considered to affect genetic structures, mainly through the expansion and contraction of organismal distributions and through migrations from the Asian continent across land bridges during the Quaternary (Millien‐Parra & Jaeger, 1999; Motokawa, 2017; Qiu, Fu, & Comes, 2011). In this context, the geohistory of Japan, along with biological events such as distribution shifts, isolation, and divergence of populations, is probably engraved within organismal genomes. Most of these phylogeographical studies on animal species were conducted for vertebrate and insect species. However, phylogeography of several other animal species, including terrestrial species of the phylum Annelida, is poorly understood.

**Figure 2:**
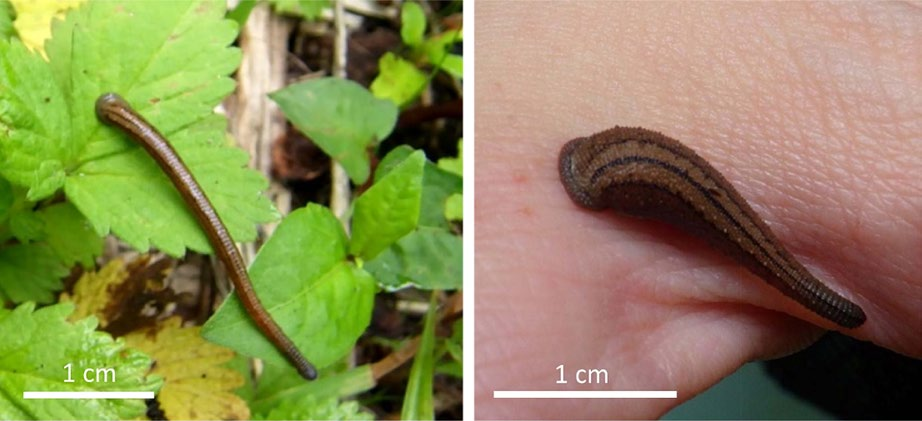
*Haemadipsa japonica* photographed in Niigata Prefecture

Leeches are segmented worms that belong to the class Clitellata, phylum Annelida (Rousset, Plaisance, Erseus, Siddall, & Rouse, 2008), and that differ from other annelids by having two (oral and caudal) suckers. The family Haemadipsidae, whose members are known for their blood‐feeding habits, comprises <70 species and is highly diverse, especially in tropical regions with high humidity in Indo‐Pacific rainforests (Borda & Siddall, 2010). The genus *Haemadipsa*, a member of Haemadipsidae, is very abundant in Southeast Asia (Won et al., 2014). *Haemadipsa japonica *Whitman (Haemadipsidae) is endemic to Japan (Figures 1 and 2); it is distributed in Honshu, Shikoku, and Kyushu (Aizawa & Morishima, 2018; Borda & Siddall, 2010; Whitman, 1886). *Haemadipsa japonica* inhabits the litter of the temperate evergreen and temperate deciduous forests, and it feeds on blood from host mammals, such as sika deer (*Cervus nippon*), wild boar (*Sus scrofa*), Japanese serow (*Capricornis crispus*), and humans (Sasaki, Saito, & Harada, 2005; Sasaki & Tani, 2008; Sugiyama & Sakaniwa, 2010). As leech species have low mobility (Trontelj & Utevsky, 2012), *H*. *japonica* is expected to exhibit a clear genetic structure and phylogeographical history as previously inferred for Japanese animal species.

A previous study using mitochondrial and nuclear DNA sequences of leech species revealed that the phylogeographical history of three medicinal leeches has been shaped by population differentiation occurred during a postglacial range expansion and by present climatic influences (Trontelj & Utevsky, 2012). However, the lack of phylogenetic and genetic structure of two of these leech species did not allow the complete understanding of their phylogeographical history. Trontelj and Utevsky (2012) then noted the need for an analysis using microsatellites exhibiting high levels of polymorphism. The use of nuclear microsatellites (nSSR) in combination with mitochondrial DNA (mtDNA) sequences for studies on leech species provides an opportunity to track the phylogeographical history, as previously reported for other animal species (e.g., Konishi, Hata, Matsuda, Arai, & Mizoguchi, 2017; López‐Uribe, Cane, Minckley, & Danforth, 2016; Michaelides et al., 2015; Rutkowski et al., 2015; Shoda‐Kagaya et al., 2010). However, few genetic analyses on leech species using nSSR markers have been conducted (e.g., Liu et al., 2016). Therefore, in the present study, we aimed to reveal the genetic structure and phylogeographical history using nSSR markers and mtDNA sequences for *H*. *japonica*.

## 2 MATERIALS AND METHODS

### 2.1 Sample collection and DNA extraction

Several studies in Japan have investigated the distributions of *Haemadipsa japonica* from the mid‐Edo era (the 1770s) to the present (Aizawa & Morishima, 2018; Yoshiba, 1994). We collected 798 individuals of *H*. *japonica* from 39 populations (Table 1; Figure 1). Leech collection was performed by walking along the forest trail and allowing leeches to attach to legs or other parts. The collected leeches were stored in 99.9% ethanol. Total genomic DNA was extracted using the DNeasy Blood & Tissue Kit (Qiagen, Hilden, Germany). Tissues from the caudal sucker were used for DNA extraction to prevent contamination from residual blood from recent feedings.

**Table 1:** Localities and number of samples of *Haemadipsa japonica* analyzed using nuclear microsatellites (nSSR) and mitochondrial DNA (mtDNA) sequencing

Population	Prefecture	Locality	Lat. (N)	Long. (E)	Alt. (m)	*N*	
						nSSR	mtDNA
1	Akita	Gojome	39°54'14"	140°11'18"	115	19	12
2		Akita				25	16
		Mt. Manaita, Igawa	39°52'05"	140°09'58"	125	(8)	(8)
		Nibetsu	39°48'12"	140°15'43"	268	(17)	(8)
3	Iwate	Omyojin, Shizukuishi	39°38'37"	140° 53'41"	–	7	8
4	Miyagi	Kinkazan, Ishinomaki	38°17'51"	141°33'23"	134	6	6
5		Gobansho Park, Ishinomaki	38°17'15"	141°31'05"	189	16	16
6	Niigata	Sado Island, Sado	38°00'42"	138°28'22"	388	21	12
7		Haidegawa dam, Gosen	37°38'37"	139°17'36"	201	24	12
8		Hiruno, Gosen	37°38'10"	139°11'33"	162	17	12
9		Mt. Awagadake, Kamo	37°34'45"	139°08'51"	147	22	12
10	Tochigi	Mt. Takahara, Shioya	36°52'00"	139°49'00"	860	26	16
11		Fujiwara, Nikko	36°48'15"	139°42'01"	407	29	16
12		Funyu, Shioya	36°47'51"	139°48'56"	424	21	16
13		Kasuo, Kanuma	36°33'41"	139°32'55"	551	24	15
14		Mitaki, Sano	36°32'21"	139°29'03"	482	29	15
15		Akiyama, Sano	36°30'21"	139°31'44"	–	20	16
16	Gunma	Tanigawa, Minakami	36°46'53"	138°56'18"	660	22	12
17		Shima, Nakanojo	36°42'16"	138°47'30"	840	24	15
18		Mt. Myougi, Annaka	36°18'27"	138°44'03"	390	20	14
19	Chiba	Kamogawa	35°09'48"	140°08'39"	418	29	15
20	Kanagawa	Mt. Tanzawa, Kiyokawa	35°28'54"	139°11'47"	523	26	13
21	Yamanashi	Shojingataki, Hokuto	35°44'21"	138°20'09"	1,007	24	12
22	Nagano	Mt. Kazakoshi, Iida	35°31'56"	137°47'23"	723	24	12
23		Iroudo, Iida	35°23'18"	138°02'37"	709	24	12
24	Shizuoka	Misakubo, Hamamatsu	35°09'28"	137°51'48"	307	24	12
25	Gifu	Mt. Toudo, Gujo	35°44'49"	136°57'56"	–	16	16
26		Gifu				15	14
		Hachiman	–	–	–	(8)	(8)
		Yoro	–	–	–	(7)	(6)
27	Mie	Inabe				20	16
		Mt. Fujiwara	35°11'57"	136°26'37"	259	(12)	(8)
		Mt. Oike	35°11'37"	136°25'31"	545	(8)	(8)
28	Aichi	Mt. Orouiwayama, Toei	35°03'59"	137°40'03"	507	24	12
29	Kyoto	Mt. Kibune, Kyoto	35°08'19"	135°45'13"	693	19	16
30	Nara	Mt. Kasuga, Nara	34°41'23"	135°52'24"	277	24	12
31	Hyogo	Awaji Island	34°15'12"	134°52'15"	122	24	14
32		Aogaki, Tanba	35°16'39"	135°00'13"	277	20	16
33	Tokushima	Kaikawa, Naka	33°45'25"	134°14'58"	504	24	12
34	Oita	Kitagawa dam, Saeki	32°49'33"	131°37'48"	205	23	13
35	Miyazaki	Shiiba	32°21'15"	131°10'50"	942	13	13
36	Kagoshima	Mt. Shibi, Satsuma	31°59'08"	130°22'45"	572	30	16
37		Yakushima Island	30°20'00"	130°30'00"	–	23	13
38	Hiroshima	Mt. Shiraki, Hiroshima	34°30'26"	132°34'37"	271	–	2
39	Wakayama	Ueuonotaki, Kozagawa	33°42'37"	135°44'09"	382	–	1
	Total					798	503

Notes

Lat., Long., and Alt. indicate latitude, longitude, and altitude of the sampling location, respectively; *N* indicates numbers of samples analyzed for nSSR and mtDNA; numbers in parentheses indicate breakdown of pooled samples; we pooled these locations as single population because two locations were in the same mountain and/or along the same river system and because sample size of these locations was small.

### 2.2 Sequencing of mtDNA

The mitochondrial cytochrome oxidase subunit one (COI) gene sequence of 503 individuals of *Haemadipsa japonica *from 39 populations was amplified using the primers LCO1490 (5′‐GGTCAACAAATCATAAAGATATTGG‐3′) and HCO2198 (5′‐TAAACTTCAGGGTGACCAAAAAATCA‐3′; Folmer, Black, Hoeh, Lutz, & Vrijenhoek, 1994). Polymerase chain reaction (PCR) was performed in 15 µl reaction volumes containing 10 ng of genomic DNA, 1× PCR buffer, 0.2 mM of each dNTP, 1.5 mM MgCl_2_, 0.5 µM of each primer, and 0.5 U of GoTaq polymerase (Promega, Madison, WI, USA). Thermocycling conditions were as follows: an initial denaturation of 1 min at 94°C, followed by 35 cycles of 45 s at 94°C, 45 s at 50°C (annealing temperature), 1 min at 72°C, and a final extension of 10 min at 72°C. PCR products were electrophoretically separated on a 2.0% agarose gel and visualized using ethidium bromide in 1× TAE; all products exhibiting a single DNA fragment were selected for sequencing. These selected products were then purified using ExoSAP‐IT (Affymetrix, Cleveland, OH, USA). Direct sequencing of both sequence directions was conducted using an ABI PRISM BigDye Terminator version 3.1 Cycle Sequencing Kit (Applied Biosystems) on an ABI 3500 Genetic Analyzer. The obtained DNA sequences were visually inspected, including quality check, and aligned using BioEdit 7.2.5.0 (Hall, 1999). Multiple alignments of sequences were obtained using ClustalW (Thompson, Higgins, & Gibson, 1994) and then manually adjusted. All sequences were deposited in GenBank under accession numbers LC427683–LC427763.

### 2.3 Nuclear microsatellite genotyping

Nine nSSR loci, previously developed for *Haemadipsa japonica* by Morishima, Suzuki, and Aizawa (2018), for 798 individuals of leeches from 37 populations were used. Two populations—38 (Hiroshima) and 39 (Wakayama)—were excluded from this analysis because of their small sample sizes (*N* = 1–2) (Table 1). Multiplex PCR was performed in 4 µl reaction volumes containing 10 ng genomic DNA, 1× Type‐it Multiplex PCR Master Mix (Qiagen), and 0.2 µM of each primer. The PCR thermal profile was as follows: an initial denaturing for 5 min at 95°C, followed by 28 cycles of 30 s at 95°C, 90 s at 60°C, 30 s at 72°C, and a final elongation of 30 min at 60°C in the GeneAmp 2720 PCR System (Applied Biosystems, PE Corp., Foster City, CA, USA). The forward sequence of each primer pair was labeled with a fluorescent dye (FAM, NED, VIC, or PET; Table 2). Genotypes were determined using an ABI 3500 Genetic Analyzer and GeneMapper ver. 4.1 (Applied Biosystems).

**Table 2:** Characteristics of the nine nuclear microsatellite loci used for genetic analyses

Locus	Size range (bp)	*N*_A_	*H*_O_	*H*_S_	*H*_T_	*F*_ST_	*G*_ST_	*G*′_ST_	Dye	Multiplex
HJssr001	66–105	14	0.460	0.450	0.778	0.433	0.421	0.784	FAM	1
HJssr011	114–206	31	0.602	0.604	0.909	0.330	0.335	0.870	NED	1
HJssr013	117–192	23	0.596	0.608	0.900	0.335	0.324	0.841	VIC	1
HJssr014	147–206	18	0.453	0.468	0.817	0.432	0.428	0.807	PET	2
HJssr022	176–334	43	0.681	0.688	0.902	0.238	0.237	0.763	FAM	2
HJssr023	190–265	25	0.608	0.66	0.887	0.261	0.257	0.771	NED	2
HJssr026	172–326	53	0.555	0.618	0.922	0.338	0.329	0.875	FAM	1
HJssr028	217–304	27	0.642	0.643	0.889	0.277	0.277	0.785	VIC	1
HJssr029	224–255	11	0.290	0.279	0.440	0.379	0.366	0.501	NED	1
Average		27.2	0.543	0.558	0.827	0.336	0.330	0.777		

Notes

*F*_ST_, genetic difference index (Weir & Cockerham, 1984); *G*_ST_, a measure of genetic differentiation (Nei, 1973); *G*′_ST_, estimated standardized measure of genetic differentiation (Hedrick, 2005); *H*_O_, observed heterozygosity; *H*_S_, gene diversity within populations; *H*_T_, total genetic diversity over all populations; *N*_A_, number of alleles per locus; *T*_A_, annealing temperature.

* Significant deviation of the *F*_ST_ values from zero was tested using 1,000 randomizations (*p* < 0.01).

### 2.4 Data analysis for mtDNA

A phylogenetic maximum likelihood analysis of the COI haplotypes was performed using PhyML 3.0 (Guindon et al., 2010) on the web server with default settings (http://www.atgc-montpellier.fr/phyml/). The substitution model GTR+G+I was selected based on the Akaike information criterion (AIC). Bootstrap support was calculated with 1,000 replications. *Haemadipsa picta* (accession number HQ203177) was included as an outgroup based on Lai, Nakano, and Chen (2011). Phylogenetic relationships between haplotypes were reconstructed using a median‐joining network (Bandelt, Forster, & Röhl, 1999) with the NETWORK version 5.0 software (available online at: http://www.fluxus-engineering.com).

**Figure 3:**
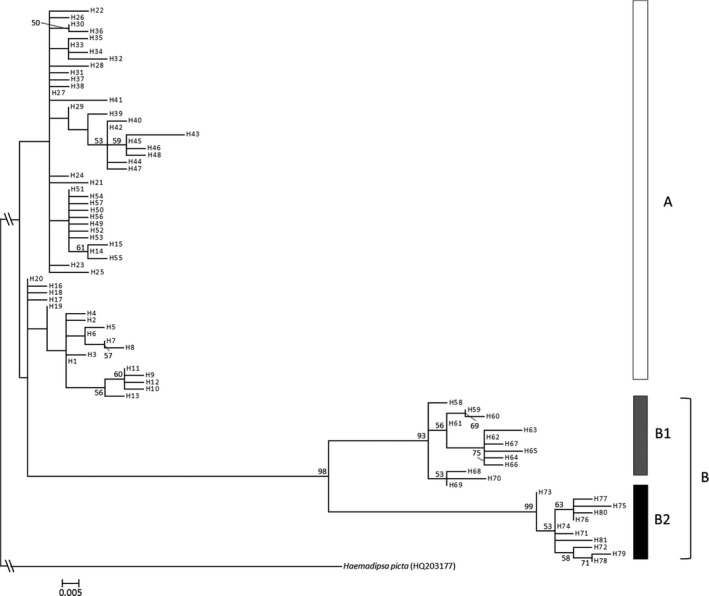
Maximum likelihood phylogeny of cytochrome oxidase subunit one (COI) haplotypes of *Haemadipsa japonica*. Only bootstrap values >50% are shown. The unit of scale bar is substitution per site

**Figure 4:**
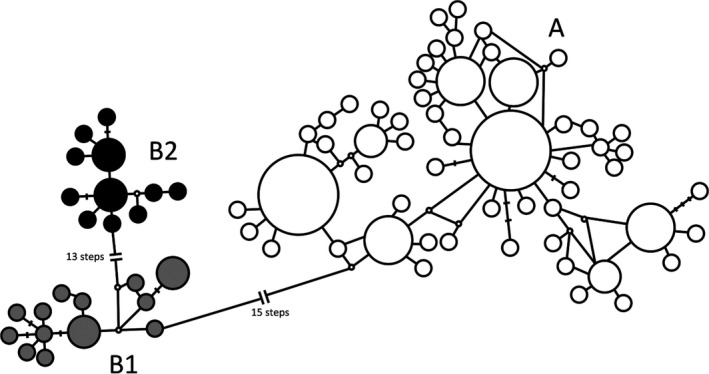
Haplotype network of cytochrome oxidase subunit one (COI) mitochondrial DNA (mtDNA) sequences of *Haemadipsa japonica*. Bars on the nodes indicate substitutions; small open circles indicate missing haplotypes; and circle sizes are proportional to the number of individuals with each haplotype. Lineage A, sublineage B1, and sublineage B2 are represented in white, gray, and black, respectively

The BEAST ver.1.8.4 (Drummond, Suchard, Xie, & Rambaut, 2012) software was used to estimate the divergence times of the mtDNA lineages (lineage A and two sublineages B1 and B2; see Results and Figures 3 and 4) of *Haemadipsa japonica* as well as other four main East Asian leech species, including *H*. *picta *(HQ203177), *H*. *ornata *(HQ203178), *H*. *rjukjuana *(HQ322462), and *H*. *montana *(HQ203182). Haplotypes of *H*. *japonica* were selected from the haplotypes with highest frequencies in each lineage, namely, H27 from lineage A, H61 from sublineage B1, and H74 from sublineage B2 (Supporting Information Appendix S1). The distribution of these leech species is as follows: *H*. *picta* is distributed in the Indochinese Peninsula, Borneo, and Taiwan (Lai et al., 2011); *H*. *ornata* is distributed in Sumatra and northeastern India (Bandyopadhyay & Mandal, 2006; Borda & Siddall, 2010); *H*. *rjukjuana* is distributed in the Indochinese Peninsula, Malay Peninsula, Indonesia, Gageodo Island of Korea, and Ryukyu Islands of Japan (Lai et al., 2011; Won et al., 2014); and *H*. *montana* is distributed in south and northeastern India (Bandyopadhyay & Mandal, 2006; Borda & Siddall, 2010). We applied the molecular calibration time to split between *H*. *ornata* and *H*. *rjukjuana* (4.47 million year; Myr; Merckx et al., 2015), using normally distributed priors with a standard deviation of 1 Myr and mtDNA COI base‐pair substitution rates of 2.5% per Myr (Kappes, 2013). A time‐calibrated Bayesian inference analysis implemented in BEAUti 1.8.4 (included in the BEAST package) was used. For the species tree prior, both of the Yule and Birth‐Death models were applied in two separate runs for comparison. Markov chain Monte Carlo (MCMC) chains were run for 10,000,000 generations and sampled every 1,000 generations. The first 1,000,000 runs were discarded as a burn‐in. We used Tracer ver.1.7 (Rambaut, Drummond, Xie, Baele, & Suchard, 2018) to verify effective sample size (ESS) values higher than 200 and to check consistency of the results. A maximum clade credibility tree was estimated with a burn‐in of 10% of the sampled trees and a posterior probability (PP) limit of 0.5 by TreeAnnotator ver. 1.8.4 (included in the BEAST package). These results were visualized using FigTree ver.1.4.3 (Rambaut, 2016).

An analysis of molecular variance (AMOVA) was conducted using Arlequin ver.3.5 (Excoffier & Lischer, 2010); populations 38 and 39 were again excluded because of their small sample sizes (*N* = 1–2). Moreover, population differentiation measures (*G*_ST_ and *N*_ST_) were estimated and the significant difference between them was tested using PERMUT (Pons & Petit, 1996) with 1,000 permutations. A significantly higher *N*_ST_ than *G*_ST_ denotes the presence of a phylogeographical structure (Pons & Petit, 1996).

### 2.5 Data analysis for nSSR

Allelic polymorphisms at each nSSR locus were evaluated. Total number of alleles detected (*N*_A_), observed gene diversity within populations (*H*_O_), gene diversity within populations (*H*_S_), total gene diversity (*H*_T_), and measures of genetic differentiation among the population—*F*_ST_ (Weir & Cockerham, 1984) and *G*_ST_ (Nei, 1973)—were calculated using the FSTAT ver. 2.932 software (Goudet, 2001). Statistical significance of *F*_ST_ was also tested using FSTAT. An estimated standardized measure of genetic differentiation (*G*'_ST_; Hedrick, 2005) was calculated using GenAlEx ver. 6.502 (Peakall & Smouse, 2006). We tested genotypic disequilibrium by using FSTAT for all pairs of loci with 1,000 permutations.

To assess population structure, an individual‐based Bayesian clustering algorithm, implemented in the STRUCTURE ver. 2.3.4 (Pritchard, Stephens, & Donnelly, 2000) software, was used. The algorithm available in the STRUCTURE software estimates allele frequencies for each gene pool (cluster) and population memberships for every individual (Hubisz, Falush, Stephens, & Pritchard, 2009). We used the LOCPRIOR model, which considers sampling information as priors (Hubisz et al., 2009), an admixture model, and the correlated allele frequency model (Falush, Stephens, & Pritchard, 2003). The STRUCTURE software was run for 100,000 MCMC iterations after a burn‐in period of 100,000 on the total dataset. STRUCTURE was run 10 times independently for each cluster (*K)* (ranging from 1 to 20). The obtained results were harvested using STRUCTURE HARVESTER (Earl & vonHoldt, 2012). The optimal number of clusters *K* was determined using two alternative approaches based on the change of mean log likelihoods of the data, LnP(D) (Pritchard et al., 2000), and rate of change in LnP(D), Δ*K* (Evanno, Regnaut, & Goudet, 2005), between successive *K* values; this is because Δ*K* is not always a good indicator of the best *K* as suggested by several studies (e.g., Janes et al., 2017; Wang, 2017). The outputs of 10 independent runs for around optimal *K* values were integrated using CLUMPP ver.1.1 (Jakobsson & Rosenberg, 2007) and visualized using DISTRUCT ver.1.1 (Rosenberg, 2004). In the STRUCTURE analyses, *F* values represent the degree of genetic drift from the ancestral population to cluster *K* and were obtained by averaging the values obtained from 10 runs at *K* = 4.

Spatial genetic structure was assessed by testing the significance of isolation by distance (IBD; Wright, 1943) using a Mantel test with 1,000 random permutations of the relationship between the matrix of pairwise *F*_ST_/(1−*F*_ST_) and that natural logarithm of geographical distance between populations (Rousset, 1997). The test was carried out using the Arlequin software. Population 26 (Hachiman and Yoro, Gifu Prefecture) was excluded from this analysis because no latitude or longitude information was available for the sampling location. We also analyzed the relationship between latitude and genetic diversity measures—expected heterozygosity (*H*_E_) and allelic richness (*Ar*)—for populations in mtDNA lineages A and B.

## 3 RESULTS

### 3.1 Genetic structure analysis for mtDNA

**Figure 5:**
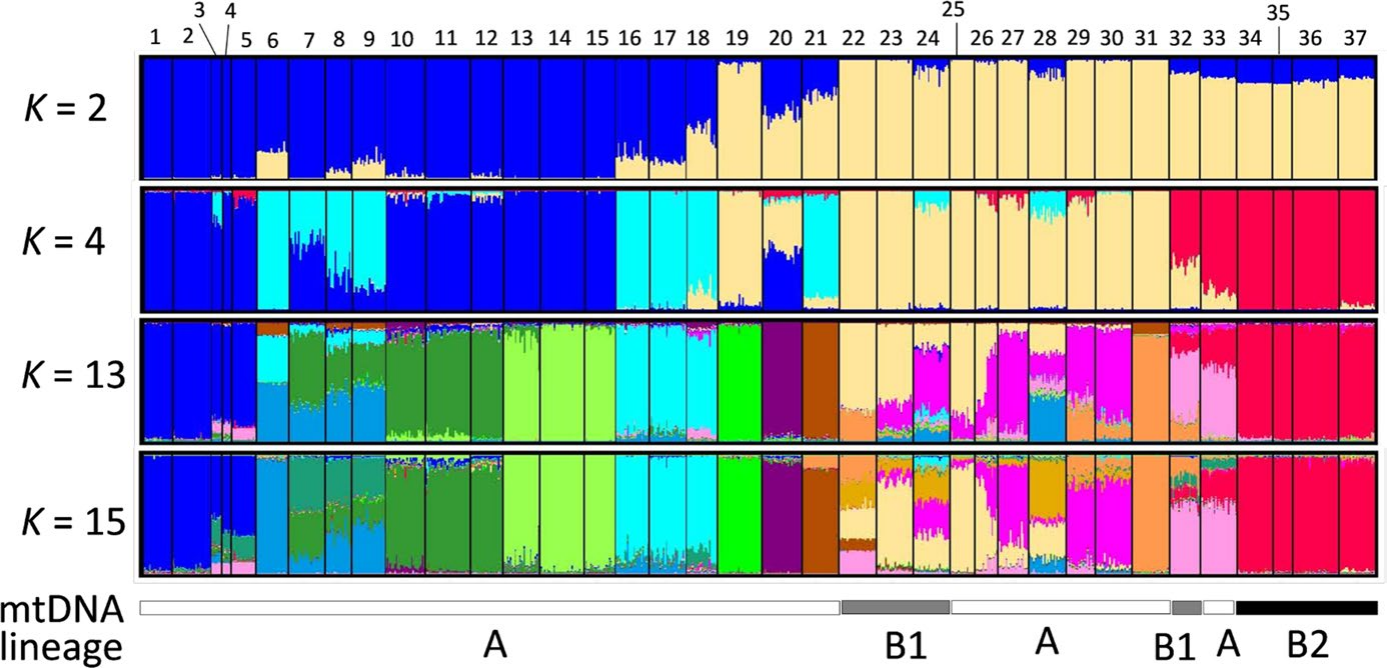
Genetic structure defined using nine nuclear microsatellite loci and the Bayesian clustering, STRUCTURE, and mtDNA lineages. Individuals are represented by vertical lines. Population codes (1–37) correspond to those shown in Table 1

Total length of the sequenced mtDNA COI fragments was 658 bp. A total of 81 haplotypes with 39 substitution sites were identified (Supporting Information Appendices S1 and S2). The maximum likelihood phylogeny indicated the existence of two different lineages with unresolved shallow polytomies (Figure 3); median‐joining haplotype network indicated the well‐resolved intraspecific phylogeny with the two different lineages (Figure 4). Lineage A was widely found across Japan (populations 1–21, 25–31, 33, and 39; Figures 1 and 5). Lineage B was divided into two sublineages, B1 and B2; B1 was found in Nagano (populations 22 and 23), Shizuoka (population 24), and Hyogo (population 32) prefectures (Figures 1 and 5); B2 was found in Kyushu (populations 34–37) and Hiroshima Prefecture (population 38; Figures 1 and 5).

**Figure 6:**
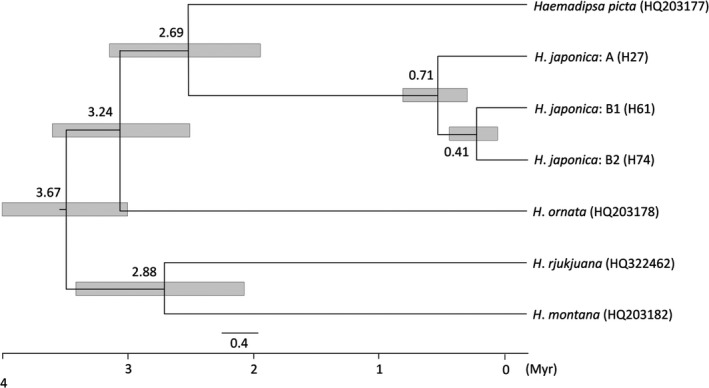
Estimation of divergence times in Haemadipsidae based on cytochrome oxidase subunit one (COI) mitochondrial DNA sequences. Clade depth indicates the mean nodal age (million years = Myr); nodes are annotated with bars indicating the 95% highest posterior density intervals for node ages. Lineage A, sublineage B1, and sublineage B2 of *Haemadipsa japonica* were obtained based on a maximum likelihood phylogeny analysis and haplotype network. The unit of scale bar is substitution per site

The results of the BEAST for *Haemadipsa japonica* and four other leech species showed a monophyletic of *H*. *japonica* and produced divergence time estimates (Figure 6). The divergence time between *H*. *japonica* and *H*. *picta* was estimated at ~2.69 million year (Myr) (95% HPD: 2.12–3.33 Myr) (Figure 6). The divergence of the two major clades (lineages A and B) of *H*. *japonica* was estimated at ~0.71 Myr (95% HPD: 0.48–0.98 Myr). Finally, the two subclades within lineage B (B1 and B2) were estimated to have diverged ~0.41 Myr (95% HPD: 0.24–0.62 Myr) (Figure 6).

The results of the AMOVA using the mtDNA data showed that the genetic differentiation between lineages A and B was highly significant (*F*_CT_ = 0.750; *p* < 0.0001; Table 3). *G*_ST_ and *N*_ST_ were 0.533 and 0.736, respectively; *N*_ST_ was significantly higher than *G*_ST_, denoting the presence of phylogeographical structure for *Haemadipsa japonica* mtDNA.

**Table 3:** Results of the analysis of molecular variance (AMOVA) using mitochondrial DNA (mtDNA) of *Haemadipsa japonica* in Japan

Source of variation	*df*	Sum of squares	Variance components	Variation (%)	*F*‐statistics
Among lineages	1	1,348.807	7.837	75.09	*F*_CT_ = 0.750*
Among populations within lineages	35	1,056.788	2.2	21.15	*F*_SC_ = 0.849*
Among individuals within populations	463	181.485	0.39	3.76	*F*_ST_ = 0.962*

Notes

mtDNA for 37 populations categorized into two distinct lineages A and B.

*df*: degrees of freedom; *F*_CT_: variation among lineages; *F*_SC_: variation among the sampled populations in each lineage; *F*_ST_: variation among sample populations among lineages.

* *P*‐values were obtained after 1,023 permutations (*p* < 0.0001).

### 3.2 Genetic diversity for nSSR

Nine nSSR loci for 37 *Haemadipsa japonica* populations showed highly variability (Table 2). The number of alleles per locus was 27.2 on average (range: 11–53); observed heterozygosity (*H*_O_), gene diversity (*H*_S_), and overall genetic diversity (*H*_T_) were 0.543 (0.290–0.681), 0.558 (0.279–0.688), and 0.827 (0.440–0.922), respectively (Table 2). The values of average genetic differentiation measures, *F*_ST_, *G*_ST_, and *G*'_ST_, were 0.336, 0.330, and 0.777, respectively (Table 2). Nonsignificant linkage disequilibrium was ascertained between all pairs of loci (*p* > 0.05).

**Figure 7:**
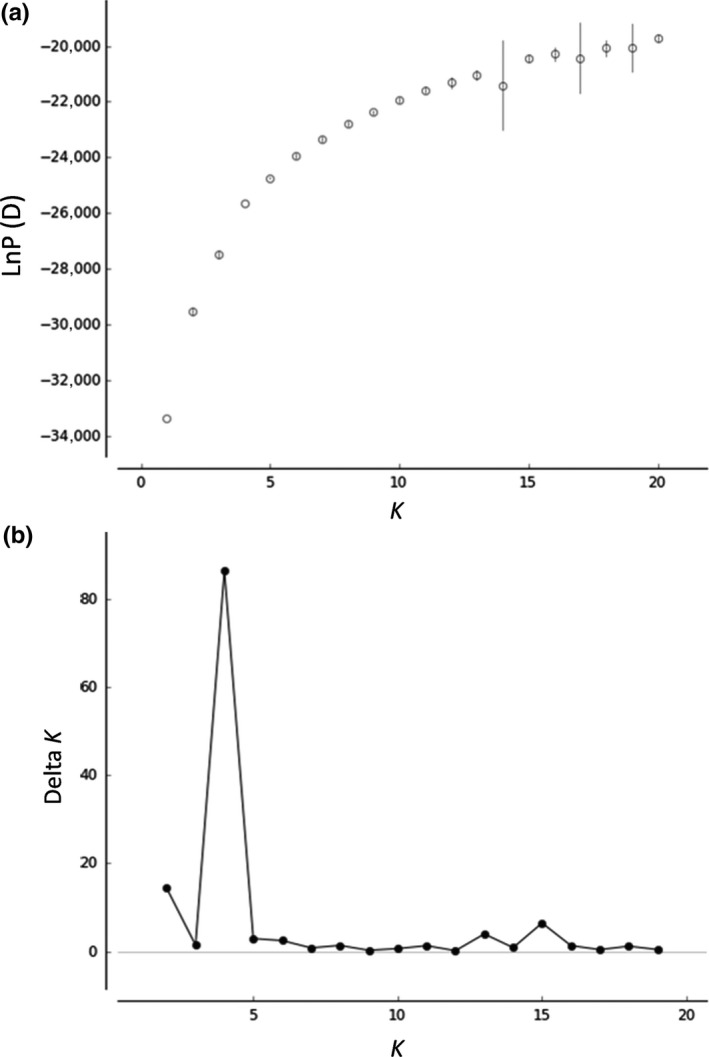
The change in the mean log likelihoods of the data, LnP(d) (a), and the rate of change in the LnP(d), *ΔK* (b), between successive *K* values obtained using the Bayesian clustering and STRUCTURE analysis for *Haemadipsa japonica*

In the STRUCTURE analysis for all 798 individuals using the nine nSSR loci, the high values of Δ*K* were observed first at *K = *4, second at *K = *2, third at *K = *15, and fourth at *K = *13 (Figure 7). The probability of the data [LnP(D)] increased progressively with each *K* and almost reached a plateau at *K = *13 (Figure 7). At *K* = 2, the 37 populations were roughly divided into two groups: populations of northeastern Japan (populations 1–18, 20, and 21) and those of southwestern Japan (populations 22–37); the observed boundary was in central Honshu. At *K* = 4, each of the two regions was roughly further divided into two; a gene pool in light blue was observed in more than half of the gene pools of each population located in central Honshu (populations 6–9, 16–18, and 21); a gene pool in red was observed in western Honshu, Shikoku, and Kyushu (populations 32–37). At *K* = 13, a geographically unique clustering pattern was observed across populations: northern Honshu (populations 1–5), Niigata (populations 6–9), northern Tochigi (populations 10–12), southern Tochigi (populations 13–15), Gunma (populations 16–18), southern Nagano (populations 22 and 23), around lake Biwa (populations 27, 29, and 30), Aogaki and Shikoku (populations 32 and 33), and Kyushu and Yakushima islands (populations 34–37). In populations 19–21 and 31, the unique gene pools were dominant (Figure 5). The results at *K* = 14 and *K* = 15 were almost the same as those observed at *K* = 13. At *K* = 4, *F* values of gene pools were 0.224, 0.177, 0.085, and 0.206 in blue, light blue, beige, and red, respectively (Figure 5).

**Figure 8:**
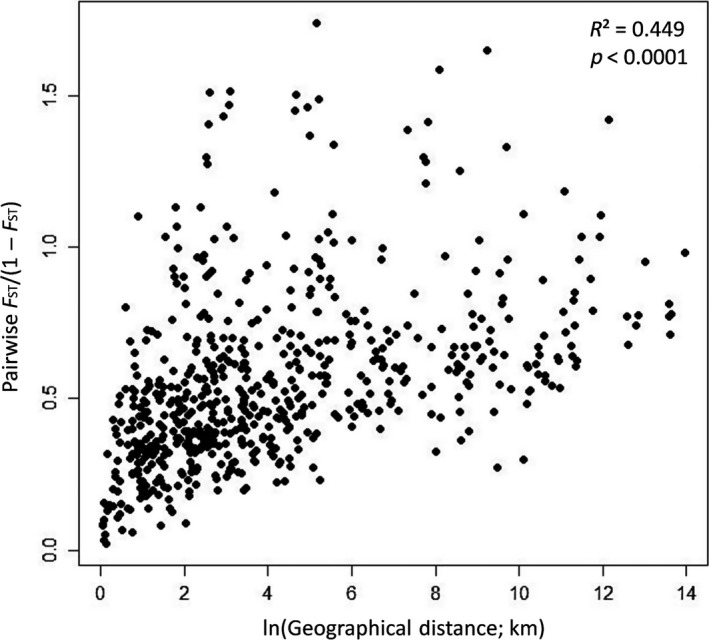
Analysis of isolation by distance (IBD) for nuclear microsatellite loci of *Haemadipsa japonica*

**Figure 9:**
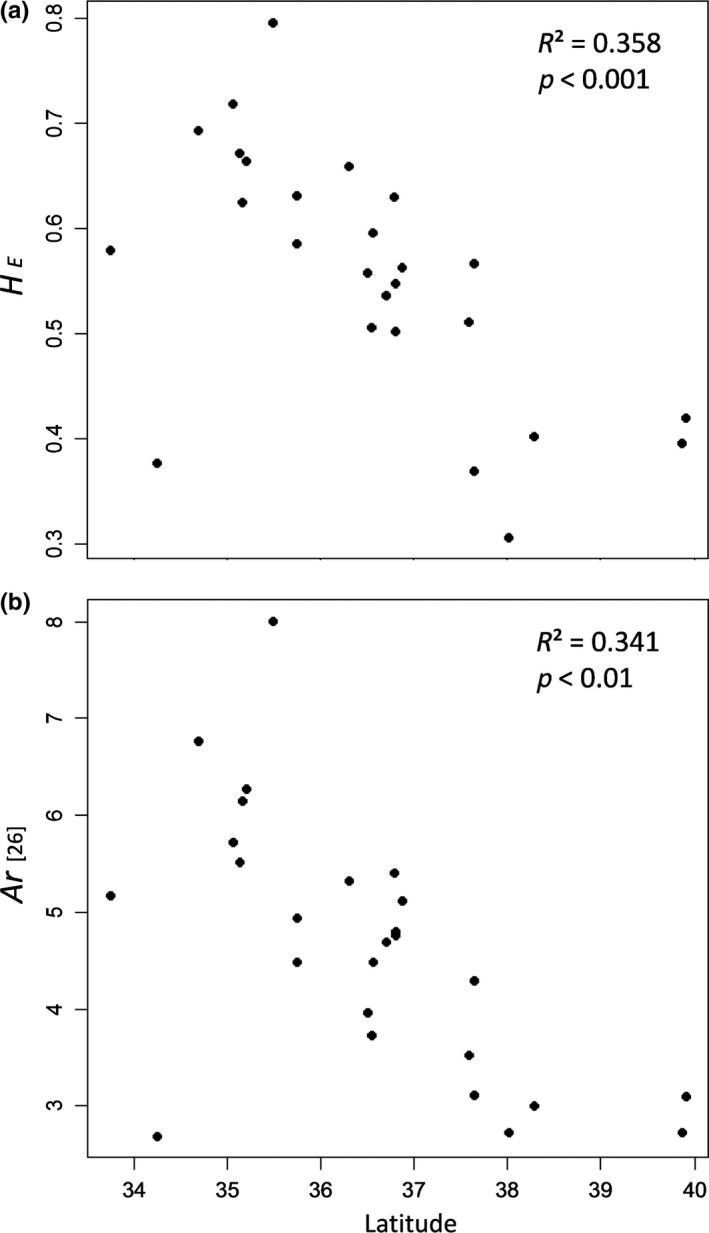
Relationship between latitude and the genetic diversity measures for nuclear microsatellite loci—expected heterozygosity (*H*_E_) (a) and allelic richness (*Ar*_[26]_) (b)—in populations of *Haemadipsa japonica* from lineage A

A Mantel test using the nine nSSR loci showed a highly significant correlation between pairwise *F*_ST_/(1–*F*_ST_) and geographical distance (*R* ^2^ = 0.449, *p < *0.0001), denoting isolation by distance for *Haemadipsa japonica* (Figure 8). The expected heterozygosity (*H*_E_) and allelic richness (*Ar *_[26]_) for all loci in each population varied from 0.306 (population 6) to 0.797 (population 20) and from 2.687 (population 31) to 8.014 (population 20), respectively (Table 4). In lineage A, these genetic diversity parameters decreased significantly with the increase in latitude (*R* ^2^ = 0.358, *p < *0.001 for *H*_E_; *R* ^2^ = 0.341, *p < *0.01 for *Ar *_[26]_; Figure 9); whereas in lineage B, the decrease in these parameters with the increase in latitude was not significant (*R* ^2^ = 0.099, *p* = 0.565 for *H*_E_; *R* ^2^ = 0.166, *p* = 0.968 for *Ar *_[26]_).

**Table 4:** Genetic diversity of *Haemadipsa japonica *populations assessed using nine nuclear microsatellite loci

Population code	*H*_E_	*Ar *[26]
1	0.420 (0.105)	3.101 (0.602)
2	0.396 (0.091)	2.726 (0.420)
3	–	–
4	–	–
5	0.402 (0.090)	3.000 (0.501)
6	0.306 (0.101)	2.734 (0.543)
7	0.369 (0.078)	3.112 (0.396)
8	0.567 (0.036)	4.294 (0.339)
9	0.511 (0.070)	3.525 (0.438)
10	0.564 (0.081)	5.127 (0.719)
11	0.548 (0.082)	4.809 (0.707)
12	0.503 (0.079)	4.766 (0.848)
13	0.597 (0.084)	4.485 (0.693)
14	0.507 (0.102)	3.733 (0.660)
15	0.558 (0.091)	3.967 (0.635)
16	0.631 (0.075)	5.406 (0.711)
17	0.537 (0.065)	4.693 (0.507)
18	0.660 (0.092)	5.326 (0.721)
19	0.626 (0.089)	6.160 (0.936)
20	0.797 (0.043)	8.014 (0.722)
21	0.632 (0.084)	4.940 (0.666)
22	0.399 (0.076)	2.868 (0.331)
23	0.615 (0.083)	6.178 (0.729)
24	0.758 (0.043)	7.344 (0.788)
25	0.586 (0.050)	4.495 (0.478)
26	0.721 (0.043)	6.162 (0.731)
27	0.665 (0.070)	6.275 (0.908)
28	0.719 (0.039)	5.732 (0.610)
29	0.673 (0.048)	5.521 (0.496)
30	0.694 (0.065)	6.778 (0.971)
31	0.377 (0.075)	2.687 (0.476)
32	0.567 (0.080)	4.703 (0.759)
33	0.580 (0.067)	5.175 (0.915)
34	0.525 (0.097)	4.388 (1.116)
35	0.401 (0.092)	3.333 (0.707)
36	0.547 (0.114)	5.426 (1.205)
37	0.550 (0.106)	5.811 (1.487)

Notes

Population codes correspond to those shown in Table 1.

*π*, nucleotide diversity; standard errors are shown in parentheses; *Ar *_[26]_, allelic richness based on the minimum sample size of 13 diploid individual; *H*, haplotypic diversity; *H*_E_, expected heterozygosity.

## 4 DISCUSSION

### 4.1 Genetic differentiation of *Haemadipsa japonica* in Japan

The genetic differentiation measures, *F*_ST_ and *G*'_ST_, of *Haemadipsa japonica*, estimated using nSSR, were much higher than those of other animals, including species with low dispersal potential, such as the canyon tree frog (*Hyla arenicolor*), the giant water bug (*Abedus herberti*), and the three aquatic leeches (*Whitmania pigra*, *Hirudo nipponica*, and *Poecilobdella manillensis*) (Table 5). Furthermore, the results of the STRUCTURE analysis at *K* = 13 showed a regionally unique clustering pattern with populations dominated by unique gene pools (populations 19–21 and 31) (Figure 5). This regional and strong genetic differentiation among populations, as well as the isolation by distance from *H*. *japonica* (Figures 5 and 8), may be a result of this leech species’ incapability of dispersing long distances (Borda & Siddall, 2010); this is also implied for aquatic leech species (Liu et al., 2016). Long‐distance dispersal of birds may be considered a reasonable explanation for the distribution of duognathous leech species; however, this hypothesis is not plausible because of the species’ feeding behavior and because it has only rarely been reported feeding on migratory birds (Borda & Siddall, 2010). The STRUCTURE analysis indicated that two genetically distinct populations (formed by populations 10–12 and populations 13–15) with different gene pools were found in Tochigi Prefecture (Figure 1 and *K* = 13 or 15 in Figure 5); these two populations were separated by less than 50 km. Although, in Japan, *H*. *japonica* is known to feed on blood from sika deer, wild boar, Japanese serow, and humans (Sasaki et al., 2005; Sasaki & Tani, 2008), our results suggest that *H*. *japonica* may not be capable of dispersing several tens of kilometers even if they move with assistance of these host animals. In this context, the low mobility of *H*. *japonica* may have resulted in a strong genetic differentiation and a low level of gene flow between populations.

**Table 5:** Genetic differentiation assessed using nuclear microsatellites for *Haemadipsa japonica* and other animal species

Class	Species	Genetic differentiation	Reference
Amphibia	*Ambystoma rivulare*	*F*_ST_ = 0.076	Heredia‐Bobadilla et al. (2016)
Reptilia	*Thamnophis elegans*	*F*_ST_ = 0.024	Manier & Arnold (2005)
	*Thamnophis sirtalis*	*F*_ST_ = 0.035	Manier & Arnold (2005)
Clitellata	*Haemadipsa japonica*	*F*_ST_ = 0.336	This study
	*Whitmania pigra*	*Φ*_ST_ = 0.547	Liu et al. (2016)
	*Hirudo nipponica*	*Φ*_ST_ = 0.290	Liu et al. (2016)
	*Poecilobdella manillensis*	*Φ*_ST_ = 0.118	Liu et al. (2016)
Amphibia	*Ambystoma macrodactylum*	*G*'_ST_ = 0.206	Goldberg & Waits (2010)
	*Hyla arenicolor*	*G*'_ST_ = 0.57	Mims, Kirk, Lytle, and Olden (2018)
	*Rana luteiventris*	*G*'_ST_ = 0.246	Goldberg & Waits (2010)
Insecta	*Abedus herberti*	*G*'_ST_ = 0.56	Mims et al. (2018)
Clitellata	*Haemadipsa japonica*	*G*'_ST_ = 0.777	This study
	*Whitmania pigra*	*G*'_ST_ = 0.630^a^	Liu et al. (2016)
	*Hirudo nipponica*	*G*'_ST_ = 0.284^a^	Liu et al. (2016)
	*Poecilobdella manillensis*	*G*'_ST_ = 0.216^a^	Liu et al. (2016)

Notes

*Φ*_ST_: a genetic differentiation measure analogous to *F*_ST_; *F*_ST_: genetic difference index (Weir & Cockerham, 1984); *G*′_ST_: estimated standardized measure of genetic differentiation (Hedrick, 2005).

a We calculated *G*'_ST _value of three Clitellata species based on data of Liu et al. (2016) using a formula proposed by Hedrick (2011).

### 4.2 Phylogeographical history of *Haemadipsa japonica* in Japan

Our analysis showed that *Haemadipsa japonica* was comprised of two mtDNA lineages (A and B) with no shared haplotypes within the lineage (Figures 3 and 4). The divergence of these lineages was estimated to have occurred ~700,000 years ago, and sublineages B1 and B2 were estimated to have diverged ~400,000 years ago; both estimated times are in the middle Pleistocene (Figure 6). Genetic differentiation between northeastern and southwestern lineages has been reported in several common animal species found across Honshu, Shikoku, and Kyushu, such as the Japanese sika deer (*Cervus nippon*), Japanese hare (*Lepus brachyurus*), tree frog (*Hyla japonica*), *Pelophylax* frog (*Pelophylax nigromaculatus*), and seed parasitic weevil (*Curculio hilgendorfi*) (Aoki, Kato, & Murakami, 2008; Dufresnes et al., 2016; Nagata et al., 1999; Nunome, Torii, Matsuki, Kinoshita, & Suzuki, 2010). The existence of two lineages in the aforementioned animal species was proposed based on intraspecific phylogenetic analyses, and it was tentatively explained by two biogeographical events: (a) independent migration of the two lineages from the Asian continent to Japan and (b) the expansion from northern and southern refugia within the Japanese archipelago. Considering that *H*. *japonica* and *H*. *picta* (its most closely related species) are presently not found in the Korean Peninsula nor in the continental part of northeastern China, the hypothesis of two independent colonizations from the Asian continent to the Japanese archipelago seems unlikely, although the possibility of a later extinction of these *Haemadipsa* species in the continental areas cannot be ruled out. Alternatively, the second explanation is likely plausible. The global glacial and interglacial cycles had already started during the middle Pleistocene (Hansen, Sato, Russell, & Kharecha, 2013). In addition, in the middle Pleistocene, islands from the Japanese archipelago, including Honshu, Kyushu, and Shikoku, supposedly formed a single landmass with the Asian continent through a land bridge (e.g., Ota, 1998). In this context, the divergence between lineages A and B and the subsequent divergence within lineage B may have occurred because of geographical differentiation; these, in turn, may have occurred because of range fragmentation within the landmass resulting from climatic change associated with the glacial and interglacial cycles that occurred in the middle Pleistocene. Furthermore, the result of the STRUCTURE analysis indicated that populations from sublineage B2 (populations 34–37) almost exclusively belonged to the gene pool in red (at *K* = 4, 13, and 15; Figure 5); the *F* value, an indicator of magnitude of genetic drift, of the gene pool in red at *K* = 4 is high (*F* = 0.206). Therefore, sublineage B2 may have diverged from other populations possessing lineage B haplotypes and undergone subsequent strong genetic drift and fixation of sublineage B2 haplotypes.

Although the divergence between lineages A and B was clear, the STRUCTURE analysis showed that the gene pools of nSSR in sublineage B1 were equal to those in southwestern Japan populations in lineage A (populations 22–33; Figure 5). Differences in phylogenetic patterns resulting from mitochondrial and nuclear data are commonly found and are attributed to sex‐biased asymmetrical dispersal, adaptive introgression of mtDNA, and demographic consequences (including genetic drift and range expansion; Toews & Brelsford, 2012). However, most leech species are protandrous hermaphrodites and reproduce by cross‐fertilization (Mann, 1962). Thus, sex‐biased dispersal cannot produce such geographical discordance pattern. Most studies that identified a large extant of mitochondrial and nuclear discordance have justified this difference as it being a result of selective advantage of introgressed mtDNA haplotypes (Toews & Brelsford, 2012). Considering that the populations of sublineage B1 of *Haemadipsa japonica* were geographically separated from each other and randomly distributed in certain areas of Honshu, adaptive introgression of mtDNA seems an unlikely explanation. An alternative explanation is demographic consequences. This means that, after the divergence of lineages A and B, populations possessing each haplotype may have had secondary contact with mtDNA introgressive hybridization and may have separated from each other again, which was then followed by population fragmentation and genetic drift. As previously discussed in this text, *H*. *japonica* is considered to have low mobility; therefore, subsequent gene flow among these populations could have been reduced. As the effective population size of mitochondrial genome in hermaphrodites represents only one half of a nuclear genome (Latta, 2006), the fixation of mtDNA lineages by genetic drift is more likely to occur than that of alleles of nuclear genome. Therefore, the different results for mitochondrial and nuclear data in populations of lineage A from southwestern Japan and sublineage B1 may have resulted from secondary contact with introgressive hybridization and subsequent random genetic drift and fixation. This explanation is in line with the randomly overlapping distribution of sublineage B1 in the distribution of lineage A in southwestern Japan.

In contrast to lineage A of southwestern Japan (populations 25–31 and 33), lineage A of northeastern Japan (populations 1–21) has almost exclusively unique gene pools (in blue and light blue) of nSSR, except for populations 19 and 20 at *K* = 4 (STRUCTURE analysis; Figure 5). In addition, genetic diversity of nSSR significantly decreased with the increase in latitude (Figure 9), whereas a nonsignificant change with the increase in latitude was found for lineage B. Considering that the *F* values of gene pools in blue and light blue at *K* = 4 in the STRUCTURE analysis are high (*F* = 0.224 and 0.177), the gradual decrease in genetic diversity with latitude within lineage A may have been a result of a northward migration followed by a subsequent founder event; such northward migration has also been suggested by studies on the alpine butterfly (*Erebia niponica*; Nakatani, Usami, & Itoh, 2007). A map of forested and nonforested biomes during the last glaciation maximum (LGM) (particularly between 18,000 and 24,000 years before present), which was drawn using fossil pollen and climate data (Harrison, Yu, Takahara, & Prentice, 2001; Qiu et al., 2011), suggests that the boreal forests (subalpine forest or cold temperate forests in Japan) were extensively spread across northern and central Honshu. Presently, *Haemadipsa japonica* does not occur in boreal forests. This is probably because of the low temperature, as *Haemadipsa* species feed at temperatures between 19 and 27°C (Wilson & Eisenberg, 1982) and thrive and reproduce between 21 and 32°C (Keegan, Toshioka, & Suzuki, 1968). This information, added to the genetic results herein presented, indicates that *H*. *japonica* could have migrated toward northern Japan after the LGM, probably during the Holocene, along with the increasing temperate and humidity. A rapid northward migration would be in accordance with our mtDNA ML tree and network. In the ML tree, largely unresolved shallow polytomies were found in lineage A. Similar mtDNA phylogenetic relationships with unresolved shallow polytomies were found in studies on the roe deer species (*Capreolus* spp.) in the Eurasian continent (Lorenzini, Garofalo, Qin, Voloshina, & Lovari, 2014); in contrast to the well‐resolved intraspecific phylogeny resulting from a network analysis, largely unresolved shallow polytomies were found for *Capreolus capreolus* occurring in Europe, besides a high divergence from *C*. *pygargus* occurring across the Eurasian continent. In general, these polytomies may have resulted from artifacts of the inference process, such as insufficient data or inappropriate sampling. On the other hand, they may indicate a real biological process, like in cases when the divergence of recently differentiated haplotypes is low or the genetic structure of populations is lacking because of high migration rates (Humphries & Winker, 2010; Lorenzini et al., 2014; Maddison, 1989; McCracken & Sorenson, 2005). In the case of the roe deer species, the poorly resolved phylogenetic relationships were considered to have been caused by high rates of migration among populations (Lorenzini et al., 2014). *Haemadipsa japonica* exhibits a strong regional genetic differentiation among populations; in addition, mtDNA lineage A of *H*. *japonica* showed several star‐like divergences of haplotypes along with the presence of phylogeographical structure (Figure 4). Therefore, our results suggested that the largely unresolved shallow polytomies found in lineage A may be because of a rapid northward migration with a rapid divergence of haplotypes probably during the Holocene.

Northeast/southwest divergence with a boundary in central Honshu, as found for lineage A, was also previously reported for several animals and plants from Japan (Okaura, Quang, Ubukata, & Harada, 2007; Jose‐Maldia et al., 2017; Sekiné et al., 2013; Shoda‐Kagaya et al., 2010; Suzuki et al., 2002; Watanabe et al., 2006). This phenomenon was presumably explained by the separation by the sea channel of Fossa Magna in the Miocene, or by subsequent orogenic movements that occurred during the Quaternary, which formed mountain chains over 3,000 m high and that currently run north to south in central Honshu. If northeast–southwest divergence caused by the Fossa Magna in the Miocene in fact occurred in *Haemadipsa japonica*, deep divergence should be engraved within the species’ mtDNA; however, this divergence was not found. Therefore, alternative explanations are that these mountain chains may have acted as geographical barriers to the dispersion of *H*. *japonica* or that complex geographical features in these mountain regions may have served as pocket refugia during a recurrent northward migration followed by a retreat toward south near central Honshu—traced by the presence of the gene pool in light blue in central Honshu (*K* = 4; Figure 5). After the northward migration, a strong genetic structure with unique regional gene pools would be formed within each lineage as a result of low mobility of *H*. *japonica* in each narrow region (*K* = 13; Figure 5).

## 5 CONCLUSION

In this study, *Haemadipsa japonica* was found to include two mtDNA lineages (A and B), which diverged in the middle Pleistocene. After the LGM, probably during the Holocene, *H*. *japonica* underwent a northward migration with a rapid divergence of haplotypes; the subsequent regional strong genetic structure may have been a result of the low mobility of *H*. *japonica*.

Since the 1980s, the populations of this leech have expanded, possibly because of the increase in mammal populations, and it has become a serious problem in several prefectures in Japan, as the leech has expanded to areas near human residence (Asada, Ochiai, & Yamanaka, 1995; Sugiyama & Sakaniwa, 2010). The clear genetic structure found in the present study may be used to predict ongoing or future dispersal routes of *H*. *japonica*, besides new areas of occurrence toward which leech populations may locally expand.

## CONFLICT OF INTEREST

None declared.

## AUTHOR CONTRIBUTION

K.M. and M.A. designed this study and conducted sampling in field. K.M. conducted nuclear microsatellite genotyping, sequencing of mtDNA, and analyzed data. All authors contributed to writing this manuscript.

## Data Availability

All mitochondrial DNA (mtDNA) sequences have been deposited in Genbank under the accessions numbers LC427683–LC427763; numbers of the mtDNA haplotype data of each population is in the Appendices; all data on microsatellite genotypes have been deposited in Dryad (https://doi.org/10.5061/dryad.b9v801n).
